# Supplementary material for: Remember how to use it: Effector-dependent modulation of spatial working memory activity in posterior parietal cortex
Source: PLoS One. 2020 Aug 26;15(8):e0238022. doi: 10.1371/journal.pone.0238022 (PMC7449404; doi:10.1371/journal.pone.0238022)
Supplement: S1 Table — (DOC) [file pone.0238022.s003.doc]

S1 Table

Average MNI coordinates of ROIs, +/- standard deviation.
