# Supplementary material for: Remember how to use it: Effector-dependent modulation of spatial working memory activity in posterior parietal cortex
Source: PLoS One. 2020 Aug 26;15(8):e0238022. doi: 10.1371/journal.pone.0238022 (PMC7449404; doi:10.1371/journal.pone.0238022)
Supplement: S2 Table — (DOC) [file pone.0238022.s004.doc]

S2 Table

The table shows the results of our 2x2 repeated measures ANOVAs with the factors “Task” (M2ST cs CT), “Response Modality” (manual vs. verbal) and “Load” (2 vs. 6 items), calculated across delay-related betas extracted from non-parietal ROIs.
