# Supplementary material for: Remember how to use it: Effector-dependent modulation of spatial working memory activity in posterior parietal cortex
Source: PLoS One. 2020 Aug 26;15(8):e0238022. doi: 10.1371/journal.pone.0238022 (PMC7449404; doi:10.1371/journal.pone.0238022)
Supplement: S1 Fig — Individual bars reflect across-subject averages of delay-related beta estimates +/- SEM in M2ST. Across-subject averages of fMRI-activity time-courses are shown in addition (+/- SEM [dashed lines]; vertical bars at 0s denote the onset of the delay phase of a trial). Blue and green colors denote verbal and manual response modalities of M2ST, respectively. Yellow and red colors denote verbal and manual responses in CT. Additional statistical information from a corresponding 2x2x2 repeated measures ANOVAs which was calculated across individual beta values considering the factors “Response Modality” (MOD: manual vs. verbal), “Load” (2 items vs. 6 items), and “Task” (match-to-sample task vs. control task) is provided in S2 Table. (DOC) [file pone.0238022.s005.doc]

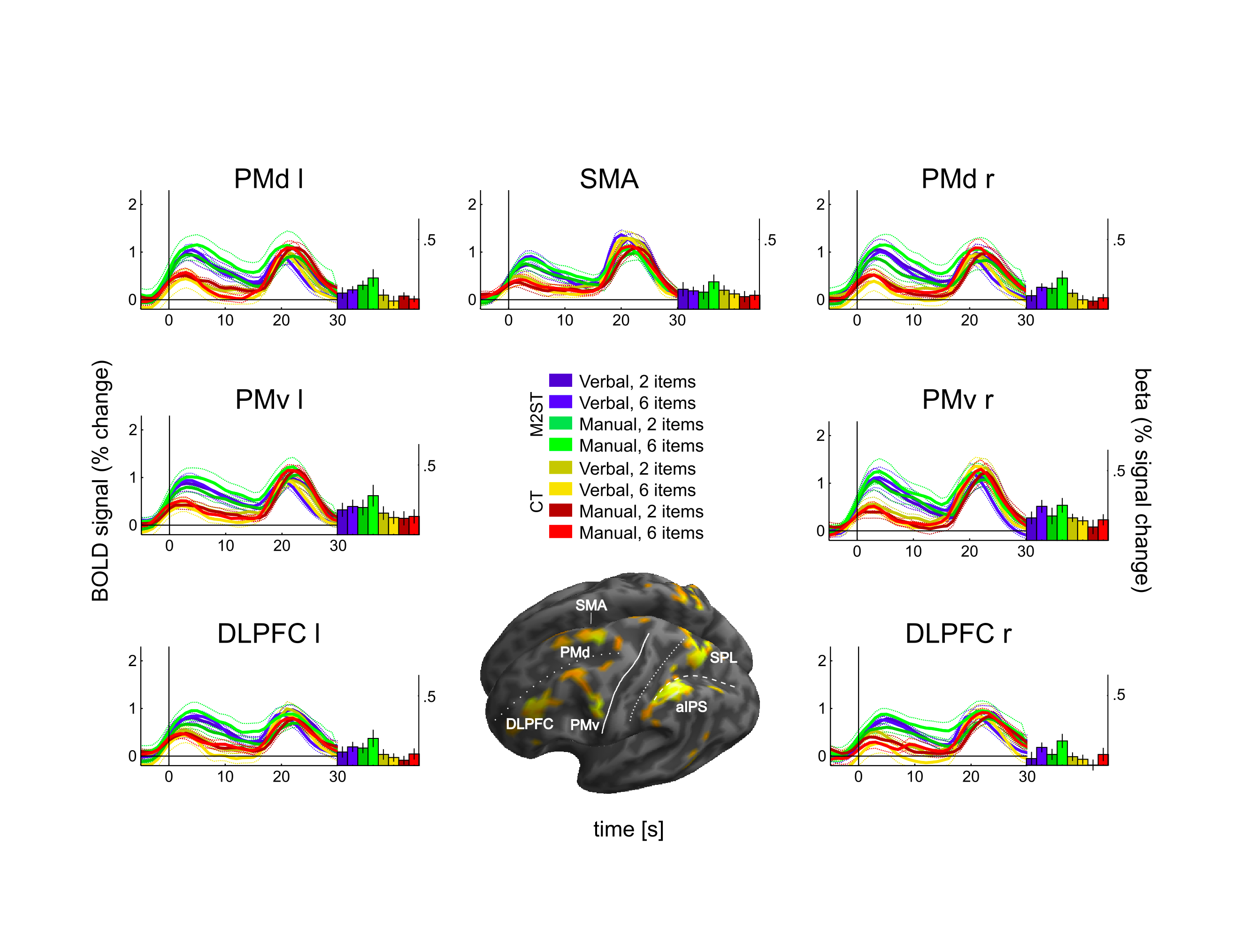


S1 Fig. WM-related brain activity in non-parietal ROIs. Individual bars reflect across-subject averages of delay-related beta estimates +/- SEM in M2ST. Across-subject averages of fMRI-activity time-courses are shown in addition (+/- SEM [dashed lines]; vertical bars at 0s denote the onset of the delay phase of a trial). Blue and green colors denote verbal and manual response modalities of M2ST, respectively. Yellow and red colors denote verbal and manual responses in CT. Additional statistical information from a corresponding 2x2x2 repeated measures ANOVAs which was calculated across individual beta values considering the factors “Response Modality” (MOD: manual vs. verbal), “Load” (2 items vs. 6 items), and “Task” (match-to-sample task vs. control task) is provided in Supplementary Table 2.
